# Supplementary material for: Hepatic SREBP signaling requires SPRING to govern systemic lipid metabolism in mice and humans
Source: Nat Commun. 2023 Aug 25;14:5181. doi: 10.1038/s41467-023-40943-1 (PMC10457316; doi:10.1038/s41467-023-40943-1)
Supplement: Supplementary file 1 — Supplementary Information [file 41467_2023_40943_MOESM1_ESM.pdf]

Hepatic SREBP signaling requires SPRING to govern systemic lipid metabolism in mice and humans

Sebastian Hendrix[1], Jenina Kingma[1], Roelof Ottenhoff[1], Masoud Valiloo[1], Monika Svecla[2], Lobke F. Zijlstra[1], Vinay Sachdev[1], Kristina Kovac[1], Johannes H.M. Levels[3], Aldo Jongejan[4], Jan F. de Boer[5,6], Folkert Kuipers[5,7], Antoine Rimbert[8], Giuseppe D. Norata[2], Anke Loregger[1,#], and Noam Zelcer[1,§]

Supplementary information

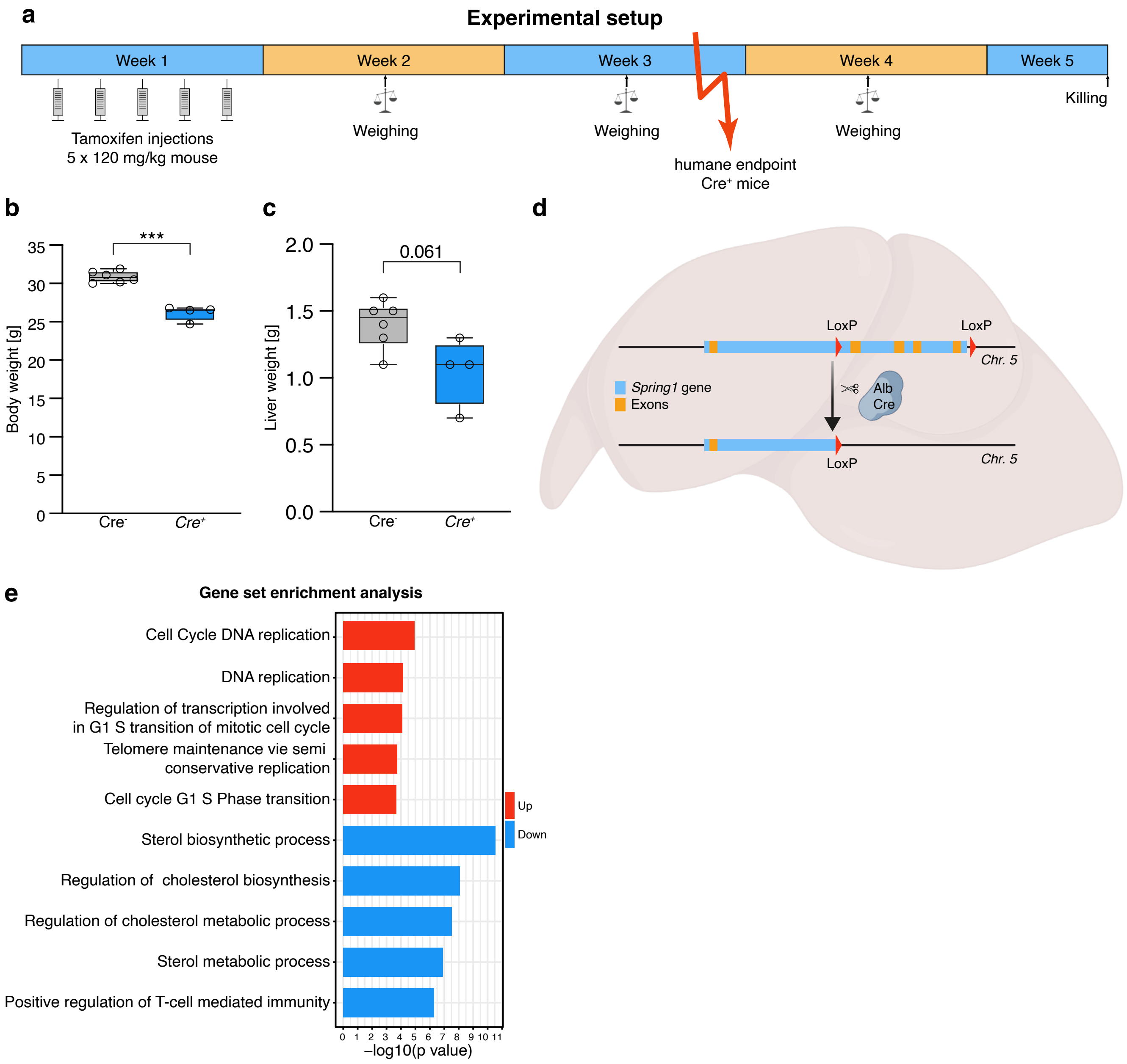

a
Experimental setup
Week 1
Week 2
Week 3
Week 4
Week 5
Tamoxifen injections
5 x 120 mg/kg mouse
Weighing
Weighing
Weighing
Killing
humane endpoint
Cre⁺ mice
b
Body weight [g]
35
30
25
20
15
10
5
0
***
Cre⁻
Cre⁺
c
Liver weight [g]
2.0
1.5
1.0
0.5
0.0
0.061
Cre⁻
Cre⁺
d
LoxP
LoxP
Chr. 5
Spring1 gene
Exons
Alb
Cre
LoxP
Chr. 5
e
Gene set enrichment analysis
Cell Cycle DNA replication
DNA replication
Regulation of transcription involved in G1 S transition of mitotic cell cycle
Telomere maintenance vie semi conservative replication
Cell cycle G1 S Phase transition
Sterol biosynthetic process
Regulation of cholesterol biosynthesis
Regulation of cholesterol metabolic process
Sterol metabolic process
Positive regulation of T-cell mediated immunity
Up
Down
0 1 2 3 4 5 6 7 8 9 10 11
−log10(p value)

Supplementary figure 1. Tamoxifen-induced global ablation of *Spring* leads to lethality. (***a***) Scheme depicting the planned experimental timeline used to assess the consequence of global *Spring* deletion. (***b,c***) Male, 8 week old *CreERT2⁻/Spring*$^{(fl/fl)}$ (Cre$^-$) and *CreERT2⁺/Spring*$^{(fl/fl)}$ (Cre$^+$) (n=6/group) were injected with the indicated dose of tamoxifen. Global loss of *Spring* in *CreERT2⁺/Spring*$^{(fl/fl)}$ required their humane endpoint sacrifice ~2.5 post injection. *Note:* 2x *CreERT2⁻/Spring*$^{(fl/fl)}$ succumbed before we were able to sacrifice them. At sacrifice (***b***) body weight (p-value: 0.0003) and *(***c***)* liver weight were determined. (***d***) Schematic illustration of the strategy used to disrupt hepatic *Spring* expression. (***e)*** Gene set enrichment analysis showing the top 5 up and down regulated pathways in livers from refed LKO vs. control mic. Box plots show the median (middle line), 25th, 75th percentile (box) and minimum and maximum values (whiskers). ****p* < 0.001 analyzed by (***b,c***) Two-tailed Welch's t-test. Source data are provided as a Source Data file.

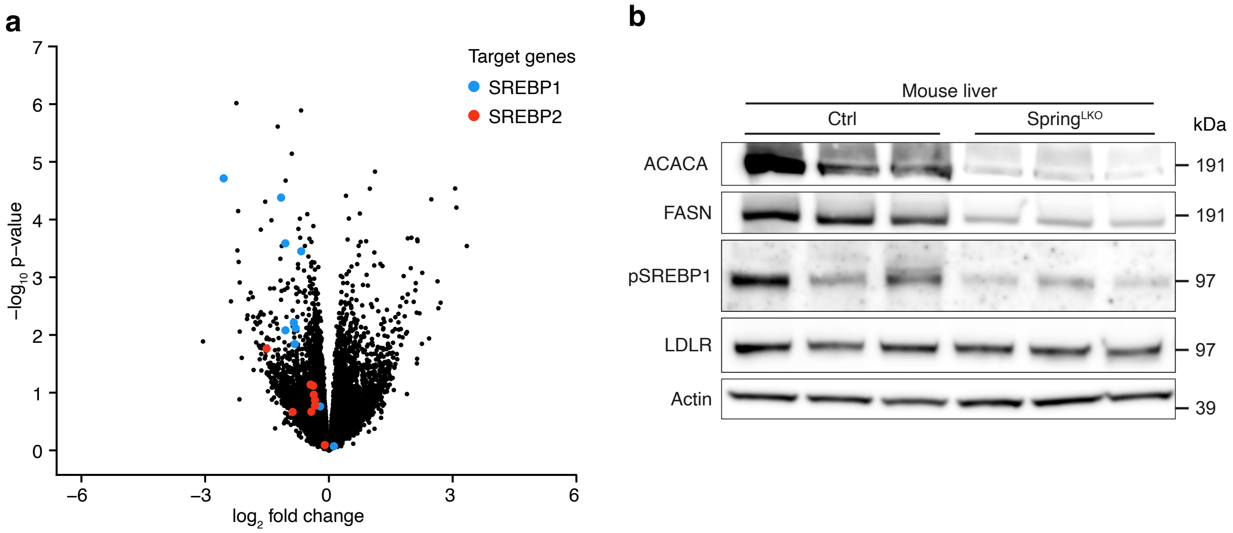
a
Target genes
SREBP1
SREBP2
−log10 p−value
log2 fold change
b
Mouse liver
Ctrl
SpringLKO
kDa
ACACA
FASN
pSREBP1
LDLR
Actin
191
191
97
97
39

Supplementary figure 2. Attenuated SREBP signaling in livers of fasted LKO mice. (***a***) (related to Figure 1) Livers were collected from 20 hours fasted control (n=9) and LKO (n=8) male mice, as described in legend of Figure 1. Transcriptional profiling of fasted livers is shown as volcano plot. Blue- and red-marked dots represent SREBP1 and SREBP2 targets, respectively. Similarly, (***b***) (related to Figure 2) liver samples from these fasted mice were immunoblotted as indicated (n=3/independent mice per genotype). pSREBP1 ; precursor SREBP1. Source data are provided as a Source Data file.

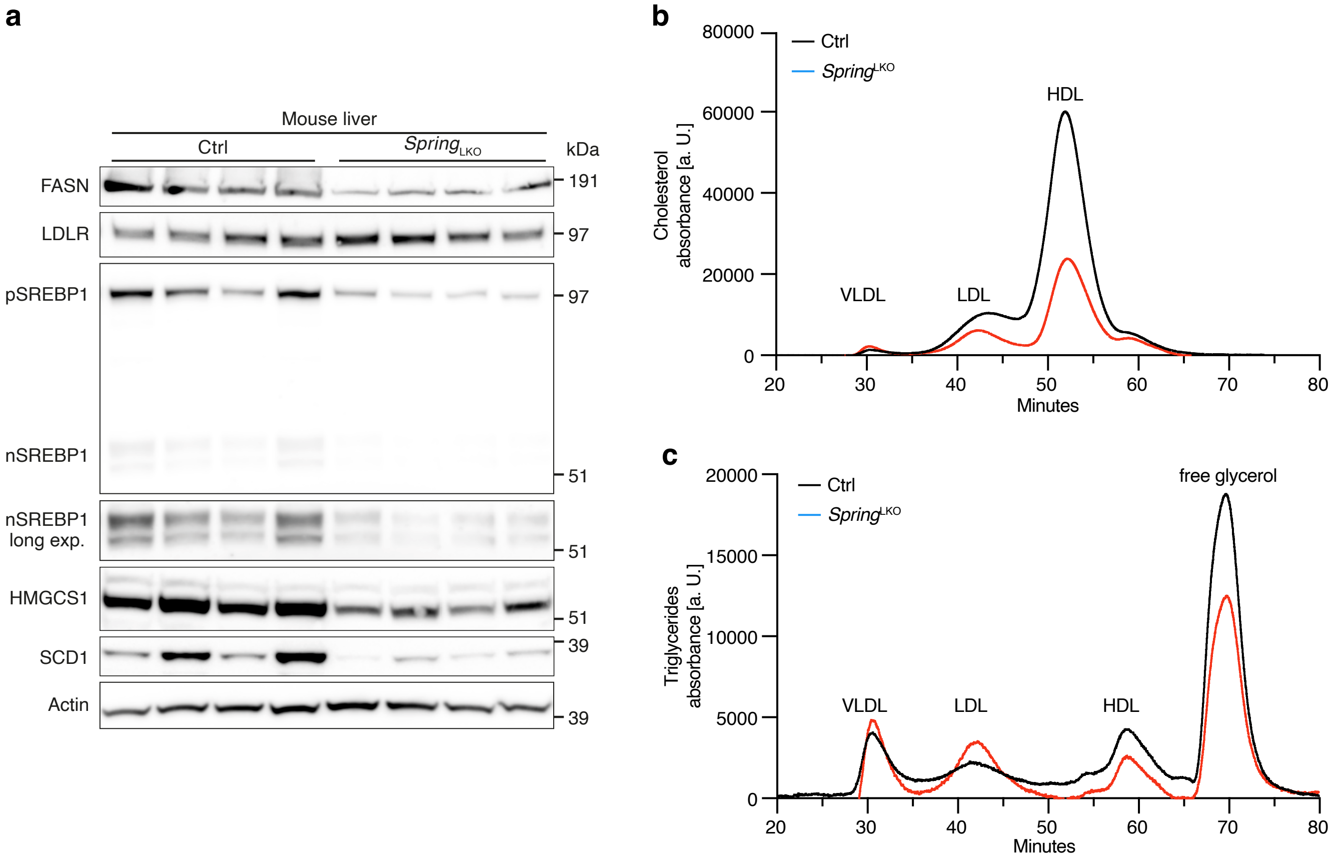
a
Mouse liver
Ctrl
Spring LKO
kDa
FASN
191
LDLR
97
pSREBP1
97
nSREBP1
51
nSREBP1
long exp.
51
HMGCS1
51
SCD1
39
Actin
39
b
80000
60000
40000
20000
0
Cholesterol
absorbance [a. U.]
Ctrl
Spring LKO
HDL
VLDL
LDL
20
30
40
50
60
70
80
Minutes
c
20000
15000
10000
5000
0
Triglycerides
absorbance [a. U.]
Ctrl
Spring LKO
free glycerol
VLDL
LDL
HDL
20
30
40
50
60
70
80
Minutes

Supplementary figure 3. Dysregulated SREBP signaling in female LKO mice. (***a***) (related to Figure 2) Liver tissue lysates from female, 8 weeks old control and LKO mice were prepared and immunoblotted as indicated (n=4/independent mice per genotype). *pSREBP1* ; precursor SREBP1, *nSREBP1* ; nuclear SREBP1. (***b,c***) (related to Figure 3) Plasma was collected from 8 week old female control and LKO mice (n=6/group). Plasma was fractionated as described in legend of Figure 3 and (***b***) total cholesterol and (***c***) triglyceride levels determined. Source data are provided as a Source Data file.

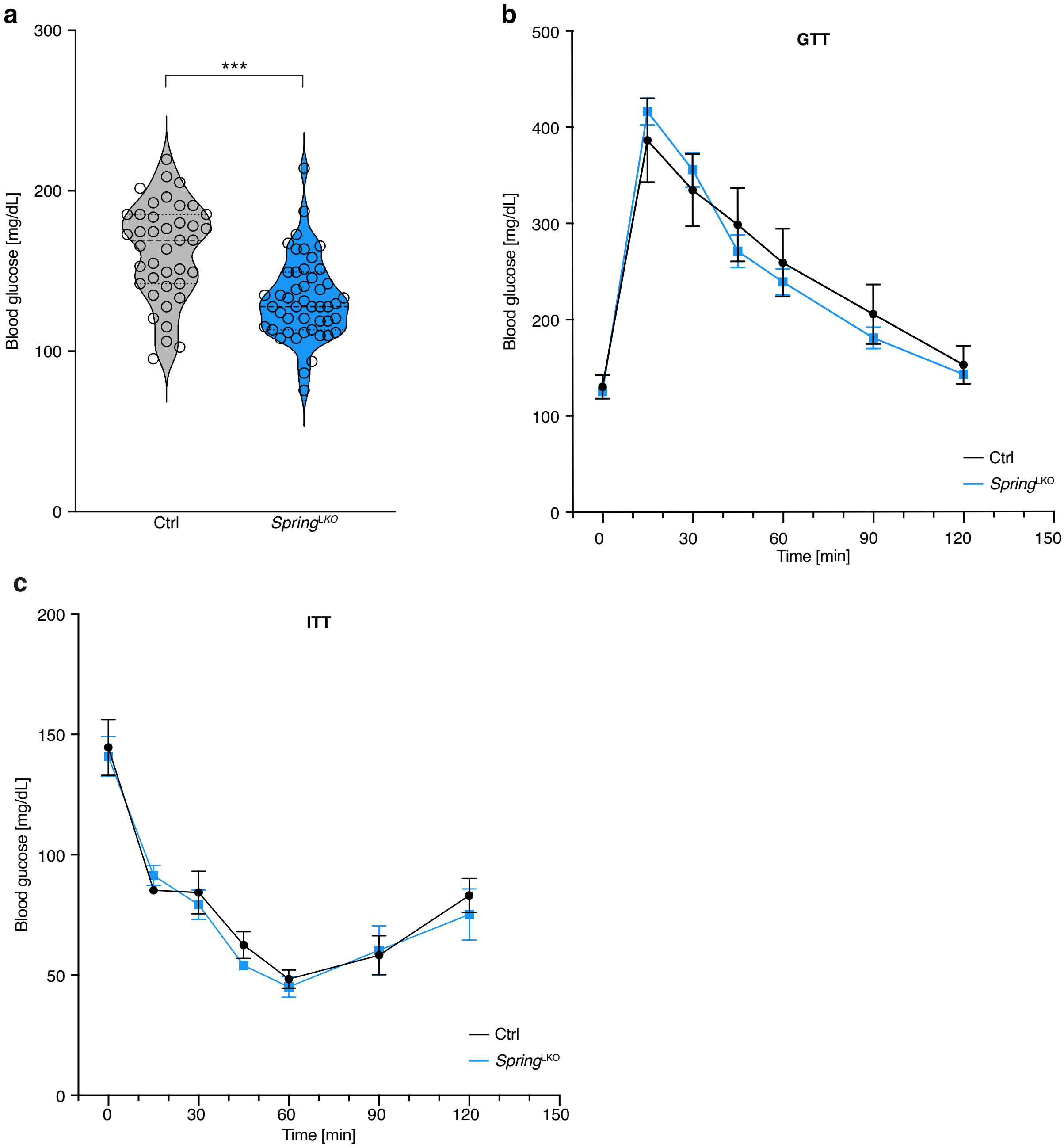

a
b
c
Blood glucose [mg/dL]
***
Ctrl
*Spring*LKO
GTT
Time [min]
Ctrl
*Spring*LKO
ITT
Blood gucose [mg/dL]
Time [min]
Ctrl
*Spring*LKO

Supplementary figure 4. Intact systemic glucose handling in LKO mice. (***a***) Blood glucose was measured in 10 week old control (n=40) and LKO (n=47) male mice and the levels shown as a violin plot with the mean and quartiles indicated. P-value: < 0.0001. (***b,c***) Male, 8 week old control and LKO mice (n=8/group) underwent a (***b***) glucose or (***c***) insulin tolerance test as described in the methods section. The concentration of glucose in blood was measured at the indicated time points. *Note:* in (***c***) 1x control and 4x LKO mice were excluded from the analysis as they became severely hyperglycemic and required injection of glucose. Each point and error represent the mean ± SEM. ***$p < 0.001$ analyzed by (***a***) Two-tailed Welch's t-test, or (***b,c***) two-way ANOVA with Holm-Sidak post hoc analysis. Source data are provided as a Source Data file.

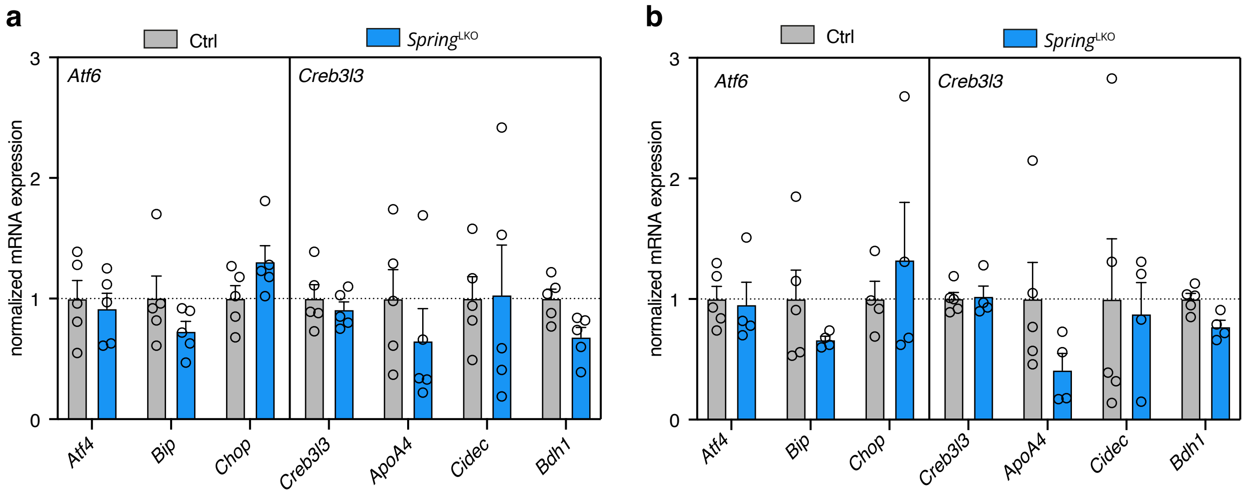
a
Ctrl
Spring^LKO
normalized mRNA expression
3
2
1
0
Atf6
Creb3l3
Atf4
Bip
Chop
Creb3l3
ApoA4
Cidec
Bdh1
b
Ctrl
Spring^LKO
normalized mRNA expression
3
2
1
0
Atf6
Creb3l3
Atf4
Bip
Chop
Creb3l3
ApoA4
Cidec
Bdh1

Supplementary figure 5. Analysis of ATF6 and CREB3L3 targets. Livers from fasted and fasted-refed mice were collected as described in Figure 1. Expression of the indicated ATF6 and CREB3L3 target genes were determined by qPCR in (***a***) fasted (n= 5 mice/group), and (***b***) fasted-refed mice (Ctrl n=5/mice; Spring^{LKO} n=4 mice). Each bar and error represent the mean ± SEM. Statistical significance was tested by (***a,b***) two-way ANOVA with Holm-Sidak post hoc analysis. Source data are provided as a Source Data file.

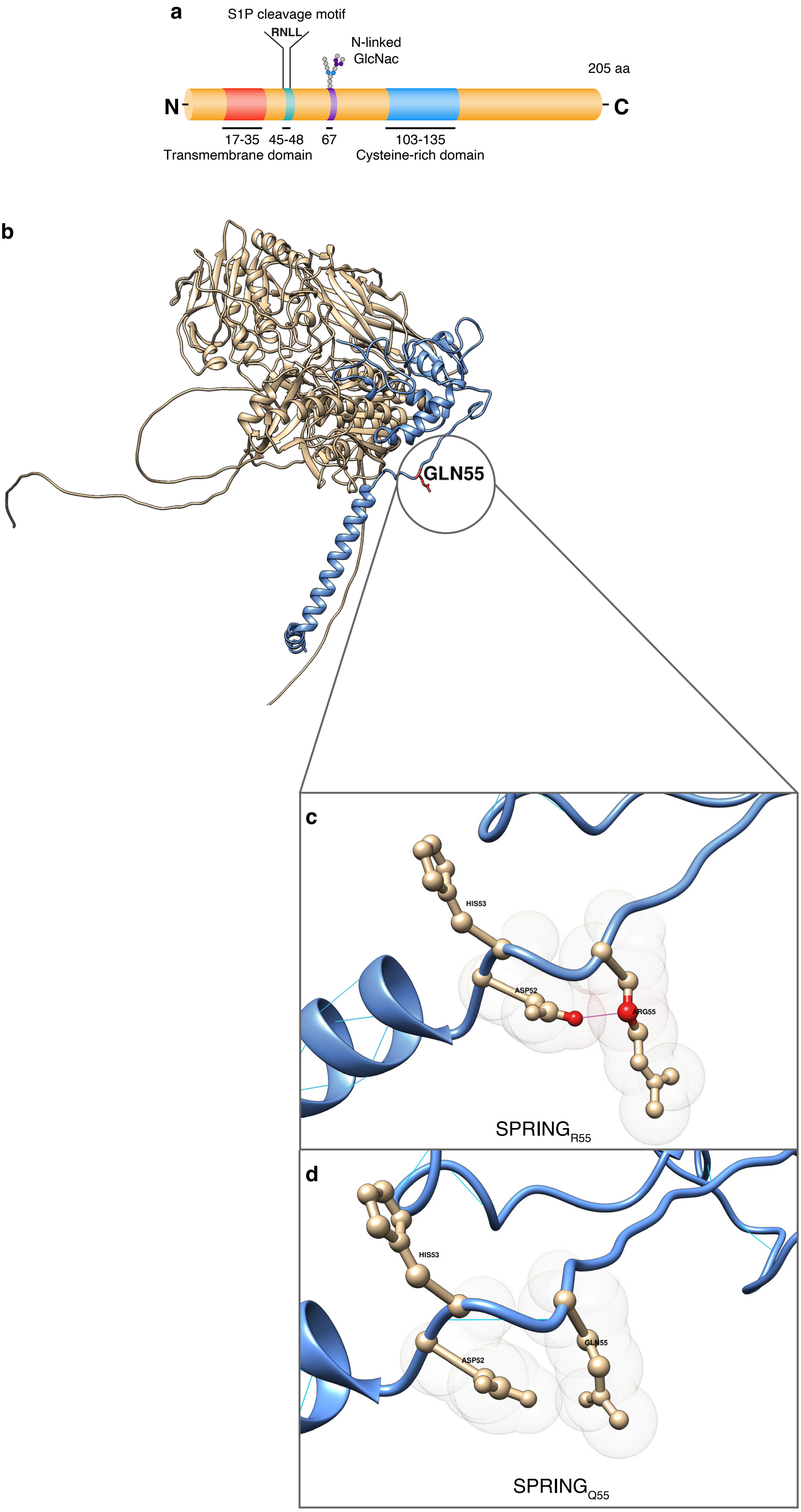
a
S1P cleavage motif
RNLL
N-linked
GlcNac
205 aa
N
C
17-35
45-48
67
103-135
Transmembrane domain
Cysteine-rich domain
b
GLN55
c
HIS53
ASP52
ARG55
SPRING R55
d
HIS53
GLN55
ASP52
SPRING Q55

Supplementary figure 6. Structural analysis of the SPRING-S1P complex. (***a***) Schematic domain structure of SPRING highlighting the S1P cleave site and the cysteine-rich domain. (***b***) Predicted Alphafold2 structure of the complex between S1P and $SPRING_{Q55}$. The top-ranked models from AlphaFold2 were used for preparing illustrations in UCSF Chimera. (***c***) Steric hinderance caused by the Q55R mutation, which results in a clash with Asp52 and loss of a hydrogen bond with His53. Steric clash is depicted in red, hydrogen bonds in light blue and van der Waals radii represented with dots (***d***) The wild type model shows no steric clashes and an intact hydrogen bond between His53 and Gln55.

Supplemental table 1: Reagents used in the study

Chemicals	Source	Identifier
**Acetic Acid [1,2-14C] Na salt**	American Radiolabled Chemicals	ACR 0173
**BSA**	Sigma	#10735086001
**Collagenase type IV**	Merck	C5138-1G
**D-glucose**	Sigma	G7528-250G
**Dexamethasone**	Sigma	D4902-100MG
**DMEM**	Invitrogen	#31966
**HBSS**	Gibco	14175-053
**Insulin-Transferrin-Selenium**	Gibco	41400045
**Mevalonate**	Sigma	#M4667
**PBS**	Fresenius Gabi	M090001/02NL
**Penicillin-Streptomycin**	Invitrogen	15140-122
**Poloxamer 407**	Merck	16758-250G
**Protease inhibitors**	Roche	#P8340
**Protease inhibitors**	Cell Signaling	5872S
**RIPA buffer**	Boston Biochem	#BP-115
**SensiFAST SYBR**	Bioline	#BIO-98020
**Simvastatin**	Calbiochem	#567021
**Sodium acetate-1-13C**	Sigma	279293-1G
**Sodium bicarbonate**	Gibco	25080094
**ß-Mercaptoethanol**	Sigma	M3148
**Tamoxifen**	Sigma	T5648-1G
**TissueTek**	Sakura Finetek	4583
**TriReagent**	Sigma	T9424
**Trypsin**	Merck	T7575-1KT

Supplemental table 2: Antibodies used in the study

Antibody target	Source	Identifier	Dilution	Used for
**LDLR**	Biovision	#3839	1:1000	WB
**SQLE**	Proteintech	#12544-1-AP	1:1000	WB
**ß-ACTIN**	EDM Millipore	#MAB1501	1:2500	WB
**ß-ACTIN**	Cell Signaling	49675	1:2500	WB
**Goat anti-Mouse IgG**	Invitrogen	A28177	1:2500	WB
**Goat anti-Rabbit IgG**	Invitrogen	A27036	1:2500	WB
**HMGCS**	Cell Signaling	422015	1:1000	WB
**SCD1**	Cell Signaling	2438S	1:1000	WB
**HMGCR**	Selfmade from A9 hybridoma cells		1:2	WB
**ACACA**	Cell Signaling	CS36765	1:1000	WB
**FASN**	Cell Signaling	CS31805	1:1000	WB
**SREBP1**	EDM Millipore	MABS1987; Clone 20B12	1:1000	WB

Supplemental table 3: qPCR primers used in the study

Gene	Species	Primer sequence
**Ldlr fw**	mus musculus	AGGCTGTGGGCTCCATAGG
**Ldlr rv**	mus musculus	TGCGGTCCAGGGTCATCT
**Sqle fw**	mus musculus	GCCTCTCAGAATGGTCGTCT
**Sqle rv**	mus musculus	CGCATCTCCCAGAATAAGGA
**Hmgcr fw**	mus musculus	TCTGGCAGTCAGTGGGAACTATT
**Hmgcr rv**	mus musculus	CCTCGTCCTTCGATCCAATTT
**Fdft1 fw**	mus musculus	TCCAAACAGGACTGGGACA
**Fdft1 rv**	mus musculus	AGACGAGAAAGGCCAATTCC
**Fasn fw**	mus musculus	GCTGCGGAAACTTCAGGAAAT
**Fasn rv**	mus musculus	AGAGACGTGTCACTCCTGGACTT
**Rplp0 fw**	mus musculus	GGACCCGAGAAGACCTCCTT
**Rplp0 rv**	mus musculus	GCACATCACTCAGAATTTCAATGG
**Rpl13a fw**	mus musculus	GGGCAGGTTCTGGTATTGGAT
**Rpl13a rv**	mus musculus	GGCTCGGAAGTGGTAGGGG
**Spring fw**	mus musculus	TCGAACTGTGCTTGGCTAAA
**Spring rv**	mus musculus	CGGTACGTGTTCTCGTGTTG
**Acaca fw**	mus musculus	GGAGATGTACGCTGACCGAG
**Acaca rv**	mus musculus	TACCCGACGCATGGTTTTCA
**Scd1 fw**	mus musculus	CCAAGCTGGAGTACGTCTGG
**Scd1 rv**	mus musculus	CAGAGCGCTGGTCATGTAGT
**Hmgcs**	mus musculus	GGTCTGATCCCCTTTGGTG
**Hmgcs**	mus musculus	TGTGAAGGACAGAGAACTGTGG
**Srebpf1c fw**	mus musculus	GGAGCCATGGATTGCACATT
**Srebpf1c rv**	mus musculus	GGCCCGGGAAGTCACTGT
**Srebp2 fw**	mus musculus	ACCTAGACCTCGCCAAAGGT
**Srepb2 rv**	mus musculus	GCACGGATAAGCAGGTTTGT
**Dhcr24 fw**	mus musculus	GGTCATGACGGACGACGTA
**Dhcr24 rv**	mus musculus	AGGGCTTGTAGTAACTGCCAAT
