# Supplementary material for: Hepatic SREBP signaling requires SPRING to govern systemic lipid metabolism in mice and humans
Source: Nat Commun. 2023 Aug 25;14:5181. doi: 10.1038/s41467-023-40943-1 (PMC10457316; doi:10.1038/s41467-023-40943-1)
Supplement: Supplementary file 3 — Description of Additional Supplementary files [file 41467_2023_40943_MOESM3_ESM.docx]

Legends for Supplementary Data 1 – 4

Supplementary Data 1 - RNAseq dataset of livers from fasted and fasted-refed LKO and control mice.

Supplementary Data 2 – Unbiased proteomics dataset of control and LKO male mice.

Supplementary Data 3 - Proteomics pathway analysis.

Supplementary Data 4 – Genetic association between SPRING and plasma lipid parameters.
