# Supplementary material for: Hepatic SREBP signaling requires SPRING to govern systemic lipid metabolism in mice and humans
Source: Nat Commun. 2023 Aug 25;14:5181. doi: 10.1038/s41467-023-40943-1 (PMC10457316; doi:10.1038/s41467-023-40943-1)
Supplement: Supplementary file 8 — Reporting Summary [file 41467_2023_40943_MOESM8_ESM.pdf]

nature portfolio

Corresponding author(s): Noam Zelcer

Last updated by author(s): Jul 30, 2023

Reporting Summary

Nature Portfolio wishes to improve the reproducibility of the work that we publish. This form provides structure for consistency and transparency in reporting. For further information on Nature Portfolio policies, see our Editorial Policies and the Editorial Policy Checklist.

Statistics

For all statistical analyses, confirm that the following items are present in the figure legend, table legend, main text, or Methods section.

n/a	Confirmed	
☐	☒	The exact sample size (*n*) for each experimental group/condition, given as a discrete number and unit of measurement
☐	☒	A statement on whether measurements were taken from distinct samples or whether the same sample was measured repeatedly
☐	☒	The statistical test(s) used AND whether they are one- or two-sided<br>*Only common tests should be described solely by name; describe more complex techniques in the Methods section.*
☒	☐	A description of all covariates tested
☐	☒	A description of any assumptions or corrections, such as tests of normality and adjustment for multiple comparisons
☐	☒	A full description of the statistical parameters including central tendency (e.g. means) or other basic estimates (e.g. regression coefficient) AND variation (e.g. standard deviation) or associated estimates of uncertainty (e.g. confidence intervals)
☐	☒	For null hypothesis testing, the test statistic (e.g. *F*, *t*, *r*) with confidence intervals, effect sizes, degrees of freedom and *P* value noted<br>*Give P values as exact values whenever suitable.*
☒	☐	For Bayesian analysis, information on the choice of priors and Markov chain Monte Carlo settings
☒	☐	For hierarchical and complex designs, identification of the appropriate level for tests and full reporting of outcomes
☒	☐	Estimates of effect sizes (e.g. Cohen's *d*, Pearson's *r*), indicating how they were calculated

Our web collection on statistics for biologists contains articles on many of the points above.

Software and code

Policy information about availability of computer code

Data collection	ImageQuant IQ800 V1.3 (Fuji), LightCycler 480 1.5.1.62 SP3 (Roche)
Data analysis	Prism Graphpad V9, ImageJ2 (V2.9.0/1.53t), ProteoWizard (version 3.0.1957), OpenMS (ver. 2.4), KNIME® (ver. 4.1.1), QIAGEN's Ingenuity® Pathway Analysis software (IPA®, QIAGEN), Trimmomatic (v0.32), HISAT2 (v2.1.0), HTSeq (v0.11.0), BiomaRt (release 94), R (v3.5.0), Bioconductor (v3.7), and the following publsihed protein prediction algorithms (SIFT, Polyphen2-HDIV, Polyphen2-HVAR, LRT, Mutation Taster, Mutation Assessor, FATHMM, PROVEAN, MetaSVM, and MetaLR). Citations are included in the manuscript for the primary publications describing the analysis packages used in this study.

For manuscripts utilizing custom algorithms or software that are central to the research but not yet described in published literature, software must be made available to editors and reviewers. We strongly encourage code deposition in a community repository (e.g. GitHub). See the Nature Portfolio guidelines for submitting code & software for further information.

Data

Policy information about availability of data

All manuscripts must include a data availability statement. This statement should provide the following information, where applicable:

- Accession codes, unique identifiers, or web links for publicly available datasets
- A description of any restrictions on data availability
- For clinical datasets or third party data, please ensure that the statement adheres to our policy

The raw proteomics data files can be accessed at the following URL: https://doi.org/10.6084/m9.figshare.22699408.v3

The raw RNAseq data files have been deposited in the Gene Expression Omnibus database under accession number GSE236045 and are available at the following URL: https://www.ncbi.nlm.nih.gov/geo/query/acc.cgi?acc= GSE236045

Datasets were released and are now publicly accessible. The URL to the Global Lipid Genetics Consortia is also indicated in the manuscript (http://csg.sph.umich.edu/willer/public/glgc-lipids2021). All other data supporting the findings of this study are available within the paper and its Supplementary Information, as also detailed in the "Data availability" section.

Research involving human participants, their data, or biological material

Policy information about studies with human participants or human data. See also policy information about sex, gender (identity/presentation), and sexual orientation and race, ethnicity and racism.

Reporting on sex and gender	n/a
Reporting on race, ethnicity, or other socially relevant groupings	n/a
Population characteristics	n/a
Recruitment	n/a
Ethics oversight	n/a

Note that full information on the approval of the study protocol must also be provided in the manuscript.

Field-specific reporting

Please select the one below that is the best fit for your research. If you are not sure, read the appropriate sections before making your selection.

☒ Life sciences ☐ Behavioural & social sciences ☐ Ecological, evolutionary & environmental sciences

For a reference copy of the document with all sections, see nature.com/documents/nr-reporting-summary-flat.pdf

Life sciences study design

All studies must disclose on these points even when the disclosure is negative.

Sample size	Sample size in in vitro experiments are detailed in the manuscript and conform to the standards in the field as to allow critical assessment of tested hypotheses. All in vitro experiments were repeated independently at least 3 times with comparable results. For the animal experiments, we based the group sample size on a power calculations using parameters established in previous studies (NQuery software).
Data exclusions	ROUT analysis (Graphpad V9) was used to identify outliers in our experiments. Identified outlier values are marked blue in the uploaded raw data file. In supplementary Figure 4C, mice were excluded (1 WT ; 4 LKO) as they developed severe hypoglycemia requiring immediate administration of glucose which prevented their inclusion in the subsequent analysis. This is clearly indicated in the accompanying figure legend.
Replication	All experiments were repeated as indicated in the manuscript, and where applicable we have now also indicated the number of independent replications. Independent experiments yielded comparable results which were aggregated and subjected to statistical significance testing. Some key measurements were also replicated by two independent scientists.
Randomization	Mouse genotypes were obtained through Hz x Hz breeding and litter mates were housed together in experiments. As such, Mendelian genetics served to randomize the groups into the studied genotypes. As the goal of these experiments was to directly compare WT vs LKO mice this was sufficient.
Blinding	Blinding was applied to the quantification of the neutral lipid images in Figure 5 and to the measurement of in vivo lipid synthesis in Figure 4. All other experiments were conducted by non-blinded scientists, as they were directly involved in the experimental design/execution and it would be unfeasible to conduct the analysis in a blinded manner.

Reporting for specific materials, systems and methods

We require information from authors about some types of materials, experimental systems and methods used in many studies. Here, indicate whether each material, system or method listed is relevant to your study. If you are not sure if a list item applies to your research, read the appropriate section before selecting a response.

Materials & experimental systems

n/a	Involved in the study
☐	☒ Antibodies
☒	☐ Eukaryotic cell lines
☒	☐ Palaeontology and archaeology
☐	☒ Animals and other organisms
☒	☐ Clinical data
☒	☐ Dual use research of concern
☒	☐ Plants

Methods

n/a	Involved in the study
☒	☐ ChIP-seq
☒	☐ Flow cytometry
☒	☐ MRI-based neuroimaging

Antibodies

Antibodies used

The antibodies used in this study are provided in separate Supp table 2. They are:

LDLR Biovision #3839 1:1000 WB
SQLE Proteintech #12544-1-AP 1:1000 WB
ß-ACTIN Merck #MAB1501; Clone C4 1:2500 WB
ß-ACTIN Cell Signaling 49675 1:2500 WB
Goat anti-Mouse IgG-HRP Invitrogen A28177 1:2500 WB
Goat anti-Rabbit IgG-HRP Invitrogen A27036 1:2500 WB
HMGCS Cell Signaling 422015 1:1000 WB
SCD1 Cell Signaling 2438S 1:1000 WB
HMGCR Selfmade from A9 hybridoma cells 1:2 WB
ACACA Cell Signaling CS36765 1:1000 WB
FASN Cell Signaling CS31805 1:1000 WB
SREBP1 EMD Millipore MABS1987; Clone 20B12 1:1000 WB

Validation

Used antibodies are listed above. These antibodies were validated by the commercial providers and additionally in our previous studies (PMID: 31117816, 31327168, 30601691, 30658189, 29903737, 28882874, 28231341 etc).

Animals and other research organisms

Policy information about studies involving animals; ARRIVE guidelines recommended for reporting animal research, and Sex and Gender in Research

Laboratory animals

In the study, male and female C57BL/6J control (SPRING fl/fl AlbCre -) and SPRING LKO mice (SPRING fl/fl AlbCre +) (own breeding) and male Rosa26-CreERT2 mice (own breeding) were used. Mice were fed a standard chow diet or high-fructose diet and housed in a temperature-controlled room under a 12-hour light-dark cycle under pathogen-specific conditions. The housing conditions are also indicated in the manuscript in the "Methods"/"Mouse experiments" section.

Wild animals

No wild animals were used in this study.

Reporting on sex

Experiments were conducted in both male and female mice, and the sex is indicated in the relevant sections (e.g figures, legends etc).

Field-collected samples

No field-collected samples were used in this study.

Ethics oversight

All the described animal experiments in this study fall under approved experimental protocols from the Dutch National Committee for animal experiments (PI: NZ) and are supervised by the Institutional Ethical Committee on Animal Experimentation of the Amsterdam UMC.

Note that full information on the approval of the study protocol must also be provided in the manuscript.
