# Supplementary material for: Hepatic SREBP signaling requires SPRING to govern systemic lipid metabolism in mice and humans
Source: Nat Commun. 2023 Aug 25;14:5181. doi: 10.1038/s41467-023-40943-1 (PMC10457316; doi:10.1038/s41467-023-40943-1)
Supplement: Supplementary file 9 — Source Data [file 41467_2023_40943_MOESM9_ESM.zip › NCOMMS-23-17280B Source Data/NCOMMS-23-17280B Blots.pdf]

Source image data

Hepatic SREBP signaling requires SPRING to govern systemic lipid metabolism in mice and humans

Hendrix et al.

Figure 2d

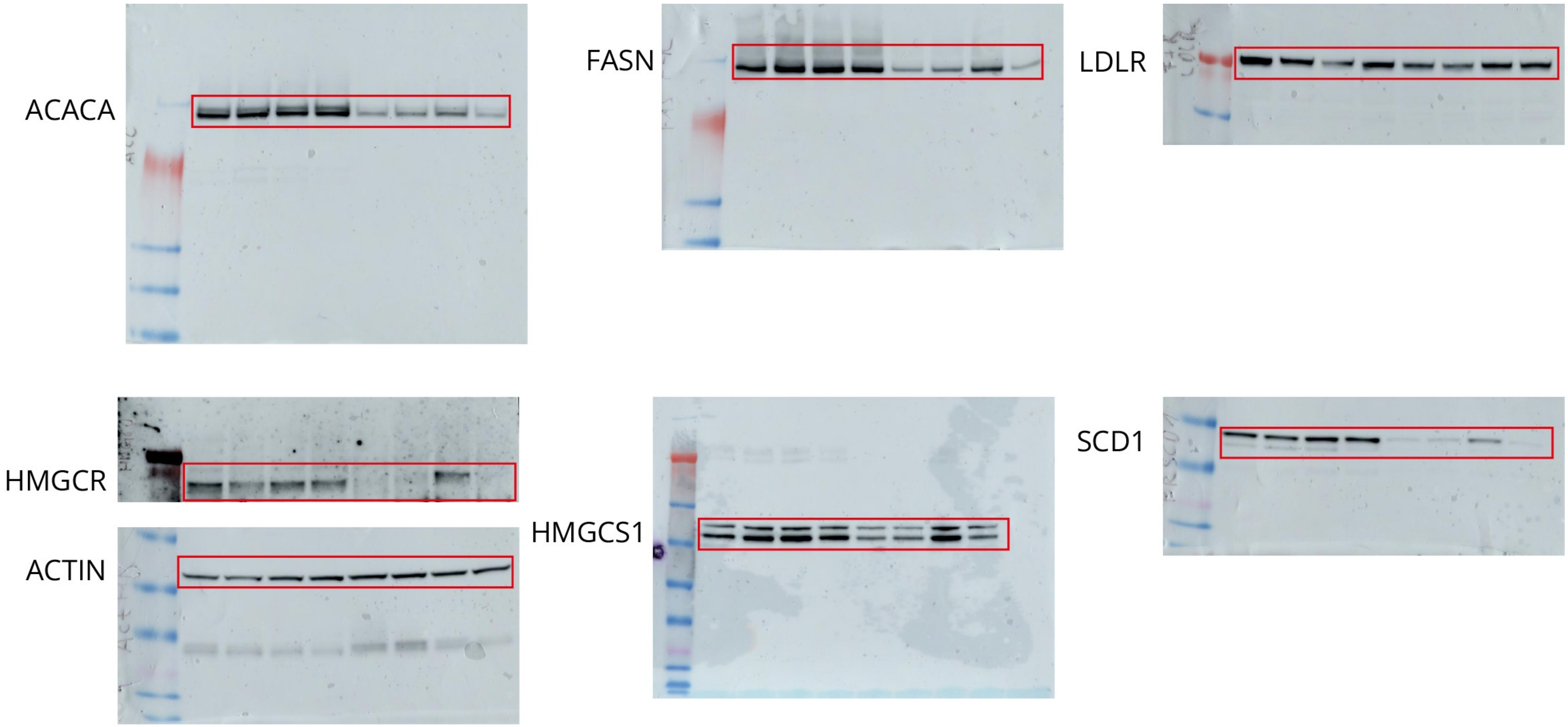

Ladder: SeeBlue™ Plus2 Pre-stained Protein Standard; Invitrogen(LC5925)

Figure 2f

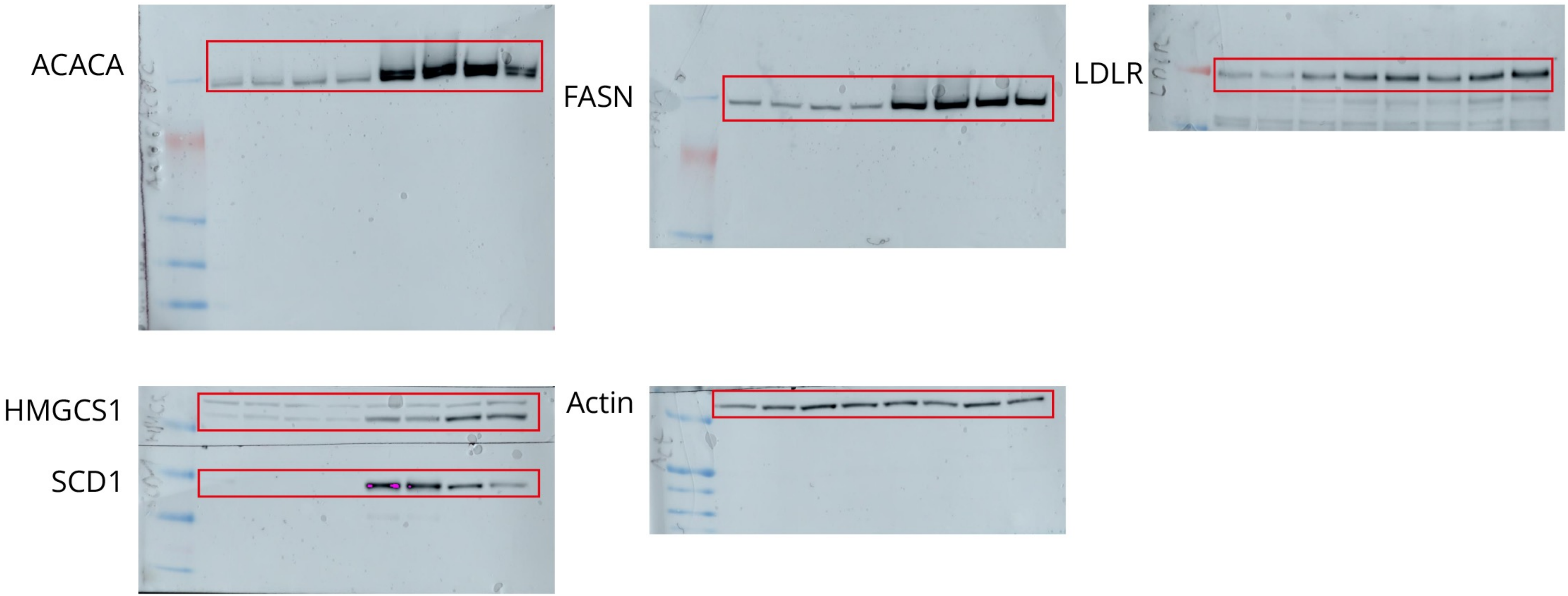

Ladder: SeeBlue™ Plus2 Pre-stained Protein Standard; Invitrogen(LC5925)

Supplementary Figure 2b

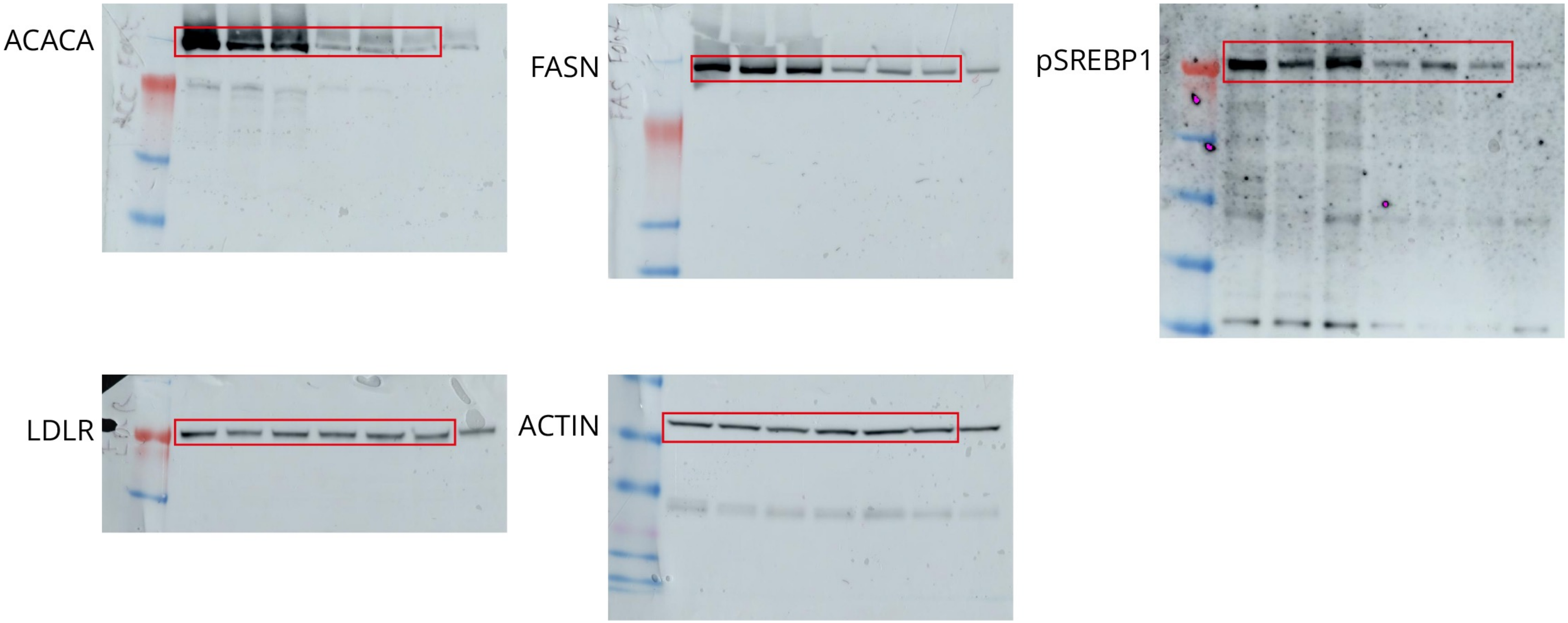

Ladder: SeeBlue™ Plus2 Pre-stained Protein Standard; Invitrogen(LC5925)

Supplementary Figure 3a

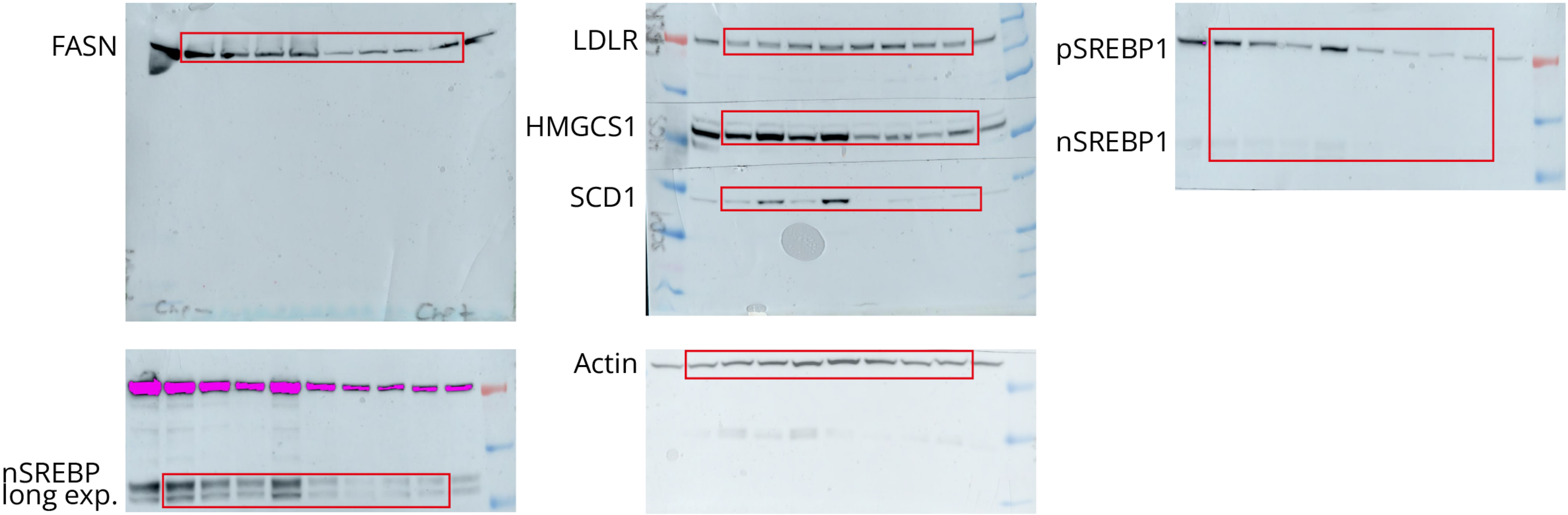

Ladder: SeeBlue™ Plus2 Pre-stained Protein Standard; Invitrogen(LC5925)
